# Antiproliferative and Proapoptotic Effects of a Protein Component Purified from* Aspongopus chinensis* Dallas on Cancer Cells* In Vitro* and* In Vivo*

**DOI:** 10.1155/2019/8934794

**Published:** 2019-01-03

**Authors:** Jun Tan, Ying Tian, Renlian Cai, Tianci Yi, Daochao Jin, Jianjun Guo

**Affiliations:** ^1^Provincial Key Laboratory for Agricultural Pest Management of Mountainous Region, Institute of Entomology, Guizhou University, Guiyang, Guizhou 550025, China; ^2^Department of Histology and Embryology, Zunyi Medical University, Zunyi, Guizhou 563000, China

## Abstract

*Aspongopus chinensis *Dallas is used as a traditional Chinese medicine. In China, clinical evidence suggests that it has anticancer activity. However, the anticancer active components are not fully elucidated. In the present study, we purified an anticancer active component (named CHP) from* A. chinensis.* To gain a comprehensive insight into the protein components, shotgun proteomic analysis was conducted. The anticancer active protein band was cut from the sodium dodecyl sulphate-polyacrylamide gel electrophoresis gel and digested with trypsin to generate peptide mixture. The peptide fragments were then analysed by liquid chromatography tandem mass spectrometry; 18 proteins were identified. In addition, we evaluated the effects of CHP on the proliferation and apoptosis of two human gastric cancer cell lines (SGC-7901 and BGC-823). The cultured cells were treated with CHP at concentrations of 20, 30, and 40 *μ*g/mL. Inhibition of cell growth was determined by the MTT assay. Hoechst 33258 staining was adopted to detect apoptosis morphologically. Apoptotic cells were quantified by Annexin V-FITC/propidium iodide staining and flow cytometry. Tumour growth was assessed by subcutaneous inoculation of 4T1 cells into BALB/c mice. There was a concentration- and time-dependent decrease in the proliferation of both cell lines at CHP concentrations of 20–40 *μ*g/mL. Apoptotic characteristics, such as karyopyknotic pyknic hyperfluorescence bolus and nuclear fragmentation, were observed in both the cell lines by Hoechst 33258 staining. Flow cytometry showed that CHP induced significant (*P *< 0.01) concentration-dependent apoptosis of SGC-7901 cells.* In vivo* assay showed that CHP can partially inhibit tumour growth derived from 4T1 cells* in vivo*. The present study is the first to report that CHP in* A. chinensis* inhibits the proliferation of cancer cell lines via the suppression of cancer cell proliferation and acceleration of apoptosis.

## 1. Introduction

Based on GLOBOCAN estimates, approximately 14.1 million new cancer cases and 8.2 million deaths occurred in 2012 worldwide [1]. Gastric cancer is the fourth major malignancy in the world and the second most common cancer in China, with approximately 679,100 new cases and 498,000 related deaths in 2015 [2], and is the third leading cause of cancer-related deaths in China [3]. Approximately 70% of patients with gastric cancer are diagnosed at an advanced stage [4]. Breast cancer is the most common malignancy in women, comprising approximately 18% of all female cancers [5]. In the past decade, the incidence of breast cancer in China increased by 27%, among which, 40% of the patients diagnosed died within five years [6].

Several chemotherapy agents offer significant advantages in the treatment of cancer. However, some of these drugs are often considered ineffective or excessively toxic. Therefore, highly effective and less toxic drugs are urgently required. During recent years, some investigators have focused on crude drugs and health products from insects, which are the most diverse and abundant of all terrestrial animals [7].

*Aspongopus chinensis* Dallas is an insect belonging to Pentatomidae and is widely distributed in China, including Guizhou, Sichuan, and Yunnan Provinces [8]. Dried* A*.* chinensis* is a common traditional Chinese medicine used to relieve pain, warm the stomach, and treat nephropathy. It is also a popular and unique food in some regions of China [9]. Extracts of* A*.* chinensis* possess strong anticancer and antibacterial activities that have been demonstrated by pharmacological studies [10, 11]. It has also been reported that* A. chinensis* has anticancer effects. Yang et al. (1999) used Xianglong powder (composed of* Pinellia ternata*,* Scolopendra subspinipes*,* A*.* chinensis*, and Rhizoma* Atractylodis Macrocephalae*) and observed its ability to induce apoptosis in human gastric cancer cells [12]. Fan et al. (2011) investigated the effect of serum containing* A. chinensis* on the expression of the apoptosis-related factors, Fas-associated death domain and p53, in human SW480 colon cancer cell line. They found that it had a significant inhibitory effect on the proliferation of SW480 and that the mechanism of inducing SW480 cell apoptosis might be associated with the stimulation of FADD and suppression of p53 [11]. These previous studies used dried* A. chinensis,* and the anticancer active components have not been fully elucidated. In the past, studies focused on the isolation and identification of small molecule natural products from dried* A*.* chinensis *[13, 14]. Four new norepinephrine derivatives, three new sesquiterpenoids, one new lactam, and twenty-three known compounds have been identified, most of which were isolated from* A*.* chinensis* for the first time. Selected members of insect-derived substances were evaluated for their biological activities against renal protection in high-glucose-induced mesangial cells and COX-2 inhibition [15]. In addition, four pairs of enantiomeric hypoxanthine analogues, two pairs of enantiomeric adenine analogues, two pyrazines, aspongpyrazines A and B, and a pair of glycerides, along with 28 known substances, were isolated from* A. chinensis*. Several new* A. chinensis*-derived natural products have been reported to promote the proliferation of neural stem cells (NSCs) [16]. However, information on the effects of peptides and proteins present in live* A*.* chinensis *is limited. Furthermore, the anticancer active components are not fully elucidated.

Our previous study results demonstrated that the anticancer active components were mainly distributed in the haemolymph and abdomen [17], and the haemolymph of* A. chinensis* can inhibit the proliferation of human gastric cancer SGC-7901 cells and human breast cancer MCF-7 cells in a dose- and time-dependent manner [18–20]. In the present study, an anticancer active component (named CHP) was isolated from the haemolymph of* A. chinensis*. The results of this study will provide a theoretical basis for investigating the anticancer effects of* A. chinensis* and support further use of this medicinal insect resource.

## 2. Materials and Methods

### 2.1. Chemicals and Reagents

The Annexin V-FITC apoptosis detection kit was purchased from Nanjing KeyGen Biotech (Nanjing, China). 3-(4,5-Dimethyl-2-thiazolyl)-2, 5-diphenyl-2H-tetrazolium bromide (MTT) was obtained from Beyotime (Beyotime Institute of Biotechnology, Shanghai, China). Dulbecco's modified Eagle's medium was purchased from Hyclone (GE Healthcare, Logan, UK).

### 2.2. Collection of Haemolymph from A. chinensis

Specimens of* A. chinensis* were caught in Zunyi, Guizhou Province, China. The specimens were authenticated by Prof. Jian-jun Guo (Institute of Entomology, Guizhou University, Guiyang, China) and washed thrice with distilled water. The samples were then dried with filter paper and the haemolymph was extracted by squeezing the insects through cheese cloth. The fluid was centrifuged at 28,000 × *g* for 10 min at 4°C and then transferred into another centrifuge tube containing the protease inhibitor phenylmethylsulfonyl fluoride (0.5 mM), lyophilised, and stored at -80°C for later use.

### 2.3. Haemolymph Purification

The haemolymph of* A. chinensis* was purified by heat treatment, ammonium sulphate precipitation, ultrafiltration, and reversed-phase high-performance liquid chromatography (RP-HPLC). Freshly prepared haemolymph was incubated at 20°C, 40°C, 60°C, 80°C, and 100°C for 30 min and then centrifuged at 28,000 × *g* for 10 min. The active anticancer components were collected, mixed with 10 mL of 10 mM Tris HCl buffer (pH 7.4), and then subjected to ammonium sulphate precipitation (0%–20%, 20%–40%, 40%–60%, 60%–80%, and 80%–100%). The pellet resulting from 40%–60% precipitation was dialysed and then concentrated and lyophilised. Three peptide fractions (> 50, 5–50, and < 5 kDa) were obtained by ultrafiltration using 5- and 50-kDa cut-off hydrophilic membranes (EMD Millipore, Billerica, MA, USA). Subsequently, the 5–50-kDa fraction was further purified by semi-preparative RP-HPLC on a C18 column (4.6 mm × 250 mm). The 5–50-kDa fraction was dissolved in solvent A (water: trifluoroacetic acid (TFA), 1000:1, v/v) and subjected to HPLC (Waters Corp., Mildford, MA, USA). The fraction (400 *μ*L) was injected and eluted with a linear gradient of 0%–30% acetonitrile at a flow rate of 0.5 mL·min^−1^. The data-processing software was Empower 2 (Waters Corp.). To isolate the active and stable components, each fraction was collected, dialysed, and lyophilised. The anticancer active protein levels were determined by Bradford method using bovine serum albumin as the standard [21].

### 2.4. Sodium Dodecyl Sulphate Polyacrylamide Gel Electrophoresis (SDS-PAGE) for Separation of Anticancer Active Components

Molecular mass of the anticancer active components was determined by SDS-PAGE as described by Laemmli [22]. SDS-PAGE was executed using an electrophoresis unit with 5% stacking and 15% separating polyacrylamide gels in 25 mM Tris HCl of pH 8.3, containing 0.18 M glycine and 0.1% SDS. The protein bands were identified by staining the gel with Coomassie Brilliant Blue R-250.

### 2.5. In-Gel Digestion

The protein band in SDS-PAGE gel was cut into a cube (approximately 1 mm × 1 mm) and washed twice with 400 *μ*L of 50 mM of NH_4_HCO_3_/ACN (50% v/v). The Cys residues were reduced by 100 *μ*L of 50 mM DTT at 56°C for 30 min and then alkylated by 100 *μ*L of 100 mM of iodoacetamide at 25°C for 30 min. After dehydration of the gel band with acetonitrile (ACN), the proteins were digested in gel overnight with 10 *μ*L of 0.05 *μ*g/*μ*L of trypsin (Promega, Madison, US) in 50 mM of NH_4_HCO_3_ at 25°C. The generated peptides were extracted with 60% ACN (v/v) in 0.1% formic acid (v/v). The peptide mixture samples were then completely dried in a vacuum centrifuge. For LC-MS/MS, the samples were reconstituted in 0.1% formic acid.

### 2.6. Protein Analysis by LC–MS/MS

We diluted each filtrate sample to 1 *μ*g/*μ*L peptide, and 5 *μ*L of each diluted sample was analysed via LC-MS/MS using the TripleTOF® 5600 mass spectrometer (AB SCIEX, Framingham, US). The mass spectrometer was coupled to an Eksigent UPLC425 system (AB SCIEX, Framingham, US). HPLC was performed with a trap and elution configuration using a C18 column (150 mm × 75 *μ*m, 3*μ*m). The LC solvents included solvent B (95% CAN + 0.1% formic acid). The sample was loaded in the trap column at a flow rate of 300 nL/min for 10 min using 100% solvent A and eluted at a flow rate of 300 *μ*L/min using a stepwise gradient (0–1 min, 5%–7% B; 1–45 min, 7%–40% B). The full-scan mass spectra obtained by the data-dependent acquisition mode were used for protein identification, and parallel reaction monitoring technique was used for quantitative analysis of the target signature peptide. The RAW data files were analysed using Protein Pilot 4.5 (AB SCIEX, Framingham, US), and high-confidence peptides were used for protein identification by setting a target false discovery rate threshold of 1% at the peptide level. Only unique peptides with high confidence were used for protein identification.

### 2.7. Cell Culture

Human gastric cancer cell lines SGC-7901 and BGC-823 and murine mammary adenocarcinoma 4T1 cells were provided by the Stem Cell Bank, Chinese Academy of Sciences. The cells were maintained as adherent monolayer cultures in DMEM medium (SGC-7901 and BGC-823 cells) and RPMI-1640 medium (4T1 cells), containing 100 U/mL penicillin, 100 *μ*g/mL streptomycin (Life Technology, Shanghai, China), and 10% foetal bovine serum (GE Healthcare, Logan, UK). The cells were cultured in an incubator with 5% CO_2_ at 37°C.

### 2.8. Cell Viability Assay

SGC-7901 and BGC-823 cells (5 × 10^3^/well) were seeded in 96-well plates and incubated for 24 h. After treatment with CHP (0, 20, 30, or 40 *μ*g/mL) for 24, 48, and 72 h, viability was determined by the MTT assay. Twenty microliters of MTT solution (5 mg/mL in phosphate-buffered saline) was added to each well, and the mixtures were incubated for 4 h at 37°C. The MTT solution was then removed and 150 *μ*L of dimethyl sulfoxide was added to the wells. The absorbance of the solution at 570 nm was measured using an automated microplate reader (Bio-Rad, Hercules, CA, US). The control values for dimethyl sulfoxide alone were subtracted from all readings and the mean absorbance was calculated. The 50% inhibition of growth (IC50) value for CHP was determined using the relative viability from the CHP concentration curve. The experiment was repeated five times. The proliferation index was calculated as the percent of viable cells in the drug-treated group versus untreated control cells using the following equation: proliferation index (%) = absorbance (treated) / absorbance (control) × 100.

### 2.9. Morphological Study

SGC-7901 and BGC-823 cells were grown on coverslips (1 × 10^5^ cells/coverslip), incubated with CHP (0, 20, 30, or 40 *μ*g/mL), and then fixed in ethanol:acetic acid solution (3:1, v/v). The coverslips were mounted on glass slides for the morphometric analysis. Three monolayers per experimental group were photographed. The morphological changes of SGC-7901 and BGC-823 cells were determined using a Nikon bright-field inverted light microscope (Tokyo, Japan) at 400× magnification.

### 2.10. Hoechst 33258 Staining

Apoptosis was analysed using the Hoechst Staining Kit (Beyotime, Shanghai, China) according to the manufacturer's protocol. The cells were grown on coverslips (1 × 10^5^ cells/coverslip) and then treated with CHP (0, 20, 30, or 40 *μ*g/mL) for 36 h. The harvested cells were washed with PBS, treated with fixing solution for 10 min, and then stained with Hoechst 33258 fluorescent dye for 5 min at 25°C. The morphological nuclear changes were observed and captured using a fluorescence microscope.

### 2.11. Cell Apoptosis by Flow Cytometry

The extent of apoptosis was analysed using the Annexin V-FITC Apoptosis Detection Kit according to the manufacturer's instructions (Nanjing KeyGen Biotech, Nanjing, China). The cells were seeded in six-well plates and then treated with CHP (10, 15, and 20 *μ*g/mL) or PBS for 24 h. The harvested cells were washed twice with ice-cold PBS and then resuspended in 500 *μ*L of binding buffer containing 5 *μ*L of Annexin V-FITC staining solution. The cells were incubated in dark for 20 min. Then, 5 *μ*L of propidium iodide staining solution was added to the cell suspension. The cells were mixed gently and incubated for 15 min in dark at 25°C. The stained cells were analysed immediately. The number of apoptotic cells was quantified using a flow cytometer (Accuri C6; BD Biosciences, San Jose, CA, US) that collected 10,000 events for analysis. The data were analysed with CFlow Plus software (BD Biosciences, San Jose, CA, US). Each assay was performed in triplicate.

### 2.12. In Vivo Assays of Antitumour Activity

Six-week-old female BALB/c mice of body weight approximately 18 g (Byrness Weil Biotech Ltd., Chongqing, China) were housed in individually ventilated cages. After approximately one week of acclimatization, the cells (1.0 × 10^6^) were resuspended in PBS (0.1 mL) and inoculated subcutaneously into the second breast of each mice. When the tumour volume reached approximately 60 mm^3^, the animals were randomised to the following treatment groups—NS (normal PBS, 0.02 mL/kg/2 d, in situ injection), CHP (40 mg/kg/2 d, in situ injection); each group contained 8 mice. Tumour length and width and animal body weight were measured every two days. The tumour volume was calculated using the following formula: (a × b^2^) / 2; where “a” is the smallest diameter and “b” is the widest one. The mice were sacrificed at the end of drug treatment and the tumours were harvested and then weighed and photographed. The* in vivo* assays were carried out in accordance with the institutional guidelines that comply with national and international laws and policies. All animal experiments were approved by the Ethics Committee of Guizhou University.

### 2.13. Statistical Analysis

All data are presented as mean ± SD. The significance of differences was evaluated by the one-way analysis of variance, followed by Fisher's least significant difference (LSD) comparison using SPSS 18.0 software (IBM, Chicago, IL, USA), The P value of <0.01 was considered statistically significant.

## 3. Results

### 3.1. Purification of Anticancer Active Component from A. chinensis

**Figure 1:**
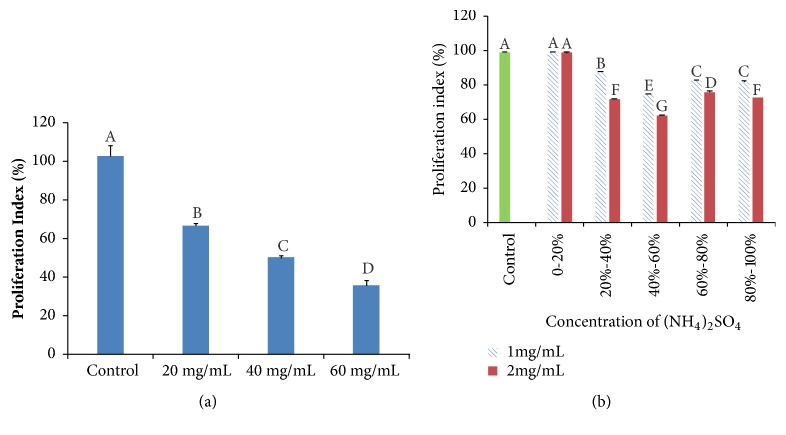
Effect of haemolymph (a) and ammonium sulphate-precipitated components (b) on the proliferation of SGC-7901 cells. The values are presented as mean ± SD of five independent experiments. Different upper case letters indicate significant difference between groups (Fisher's LSD,* P *< 0.01).

The haemolymph extracted from* A. chinensis* inhibited the proliferation of SGC-7901 cells in a dose-dependent manner (IC_50_ = 42.912 mg/mL) (Figure 1(a)). We found that when the haemolymph (2086.368 mg) was treated at high temperature, a large amount of proteins was denatured. The haemolymph treated at 60°C still exhibited anticancer activity, which was detected by the MTT assay (IC_50_ = 3.145 mg/mL) (Table 1). Therefore, the components of haemolymph treated at 60°C (named Component I) were selected for further purification.

Component I was precipitated by ammonium sulphate salting-out method, with 0%–20%, 20%–40%, 40%–60%, 60%–80%, and 80%–100% saturation gradient. The five groups of proteins were precipitated and dissolved with PBS, dialysed, and freeze-dried. The concentration of protein was determined by Bradford method and the inhibition of cell proliferation was detected by the MTT assay. The results showed that 40%–60% ammonium sulphate saturation precipitated the components that could significantly inhibit the proliferation of SGC-7901 cells (IC_50_ = 1.435 mg/mL) (Figure 1(b)). Therefore, the 40%–60% ammonium sulphate-precipitated fraction (hereafter referred to as Component II) was selected for the downstream separation and purification experiment.

**Figure 2:**
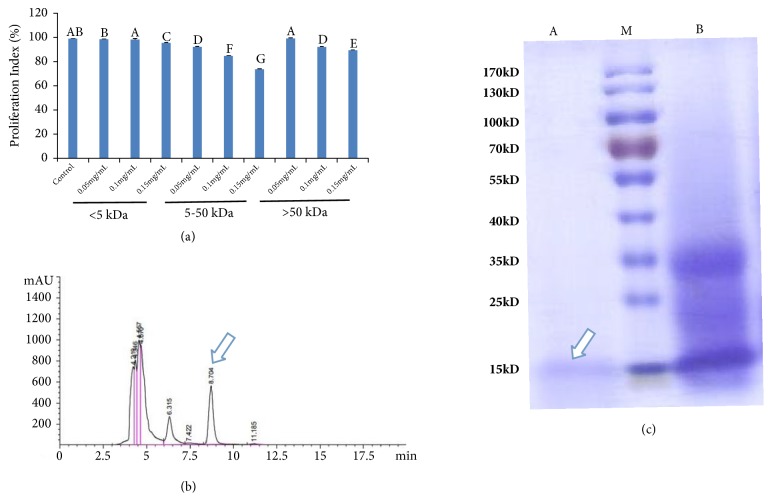
Purification of the anticancer active component. (a) Effect of three molecular weight fractions (< 5, 5–50, and > 50 kDa) on the proliferation of SGC-7901 cells. (b) Semi-preparative reversed-phase high-performance liquid chromatograms of CHP (indicated by an arrow) purified from* A. chinensis.* (c) SDS-PAGE reveals the anticancer active component. Lane A: purified proteins (CHP) (indicated by an arrow); Lane B: 5–50-kDa ultrafiltration fractions; Lane M: protein marker.

To determine the effect of molecular weight on the antiproliferative activity of proteins, Component II was fractionated to obtain three molecular weight fractions (< 5, 5–50, and > 50 kDa), which were screened for the antiproliferative activity against SGC-7901 cells. Among these fractions, the 5–50-kDa proteins showed higher potency (IC_50_ = 0.337 mg/mL) to inhibit the proliferation of SGC-7901 cells than that of the > 50-kDa (IC_50_ = 0.459 mg/mL) and < 5-kDa (IC_50_ = 4.425 mg/mL) proteins (Figure 2(a)). This indicates that haemolymph proteins of intermediate molecular weight (5–50-kDa fractions) are more effective in inhibiting the proliferation of SGC-7901 cells than smaller molecular weight proteins (< 5 kDa) and high molecular weight proteins (> 50 kDa). The 5–50-kDa fractions were fractionated on a C18 column and the protein peaks were resolved (Figure 2(b)). Finally, the anticancer active component was subjected to SDS-PAGE. A band (15 kDa) was observed in the gel (Figure 2(c)).

### 3.2. LC–MS/MS Analysis

**Figure 3:**
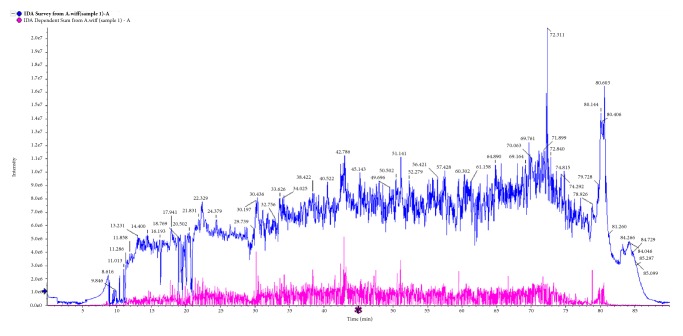
LC-MS/MS spectrogram reveals the anticancer active component (CHP).

The most active subfraction (CHP) was selected for peptide identification by LC–MS/MS. A total of 59 peptides of different lengths, ranging from 6 to 19 amino acids, were identified (Figure 3). We identified 18 proteins by LC-MS/MS analysis. Six of these had at least six unique peptides and were thus recognised with high confidence (Table 2), namely, cytochrome c (Cyt c), ferritin, superoxide dismutase, ATP synthase subunit a, NADH-ubiquinone oxidoreductase chain 1, and cytochrome b. Among these proteins, Cyt c might be a noteworthy anticancer protein, which might play the main role in anticancer effect. The release of cytochrome c from mitochondria was necessary for the formation of the Apaf-1 apoptosome and subsequent activation of caspase-9 in mammalian cells, and apoptosis was initiated [23]. This was consistent with the findings of our previous study, and the expression of caspase 3, caspase 9, and Bax proteins was significantly upregulated when SGC-7901 cells were treated with haemolymph, which indicated that apoptosis of SGC-7901 cells induced by the haemolymph might be via the mitochondrial signal pathway [24].

### 3.3. Cell Viability

**Figure 4:**
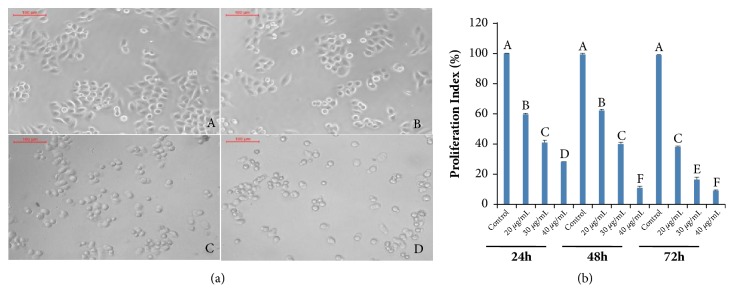
Effect of CHP on the proliferation of gastric cancer SGC-7901 cells. (a) Morphologic characterization of SGC-7901 cells treated with different concentrations of CHP (0, 20, 30, and 40 *μ*g/mL; A-D, respectively). (b) The viability of SGC-7901 cells treated with 0 (control), 20, 30, and 40 *μ*g/mL CHP. The viability of cells was determined by the MTT assay. The values are presented as mean ± SD of five independent experiments. Different upper case letters indicate significant difference between the groups (Fisher's LSD,* P* < 0.01).

**Figure 5:**
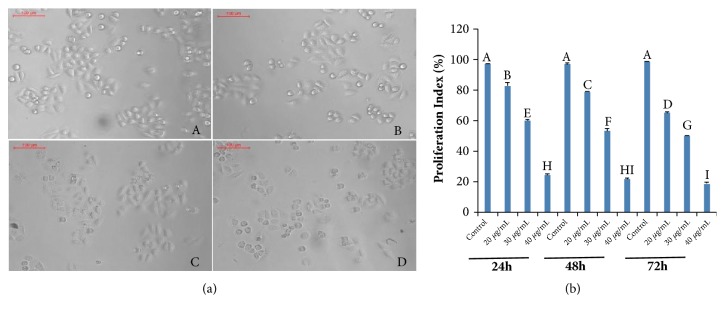
Effect of CHP on the proliferation of gastric cancer BGC-823 cells. (a) Morphologic characterization of BGC-823 cells treated with different concentrations of CHP (0, 20, 30, and 40 *μ*g/mL; A–D, respectively). (b) The viability of BGC-823 cells treated with 0 (control), 20, 30, and 40 *μ*g/mL CHP. The viability of cells was determined by the MTT assay. The values are presented as mean ± SD of five independent experiments. Different upper case letters indicate significant difference between the groups (Fisher's LSD,* P <* 0.01).

Morphological characterization of SGC-7901 and BGC-823 cells was performed after treatment with CHP. A light microscope was used to evaluate the extent of cell shrinkage at 48 h after treatment with different concentrations of the CHP protein. The results were compared with those of untreated gastric cancer cells. The most recognizable morphological changes of CHP-treated cells were cytoplasmic condensation and cell shrinkage (Figures 4(a) and 5(a)).

To determine the antiproliferative effects of CHP on gastric cancer cells, their viability was determined by the MTT assay. The MTT assay was used as a sensitive and reliable colorimetric assay to measure the viability and proliferation of cells. The cleavage of MTT has several desirable properties for assaying cell survival and proliferation. MTT is cleaved by all living, metabolically active cells, but not by dead cells or erythrocytes. The amount of formazan generated is directly proportional to the cell number over a wide range, using a homogeneous cell population. Activated cells produce more formazan than the resting cells, which enable the measurement of activation even in the absence of proliferation. These properties are all consistent with the cleavage of MTT only by active mitochondria [25]. The MTT assay was performed to determine the IC_50_ of CHP in SGC-7901 and BGC-823 cell lines. The viability of gastric cancer SGC-7901 and BGC-823 cells treated with different concentrations of CHP (0, 20, 30, and 40 *μ*g/mL) for 24, 48, and 72 h is shown in Figures 4(b) and 5(b). The treated cells showed a reduced proliferation capacity in a concentration-dependent manner (*P *< 0.01), although SGC-7901 (IC_50_ = 25.462 *μ*g/mL) was more sensitive than BGC-823 (IC_50_ = 29.003 *μ*g/mL) cells.

### 3.4. Hoechst 33258 Staining

**Figure 6:**
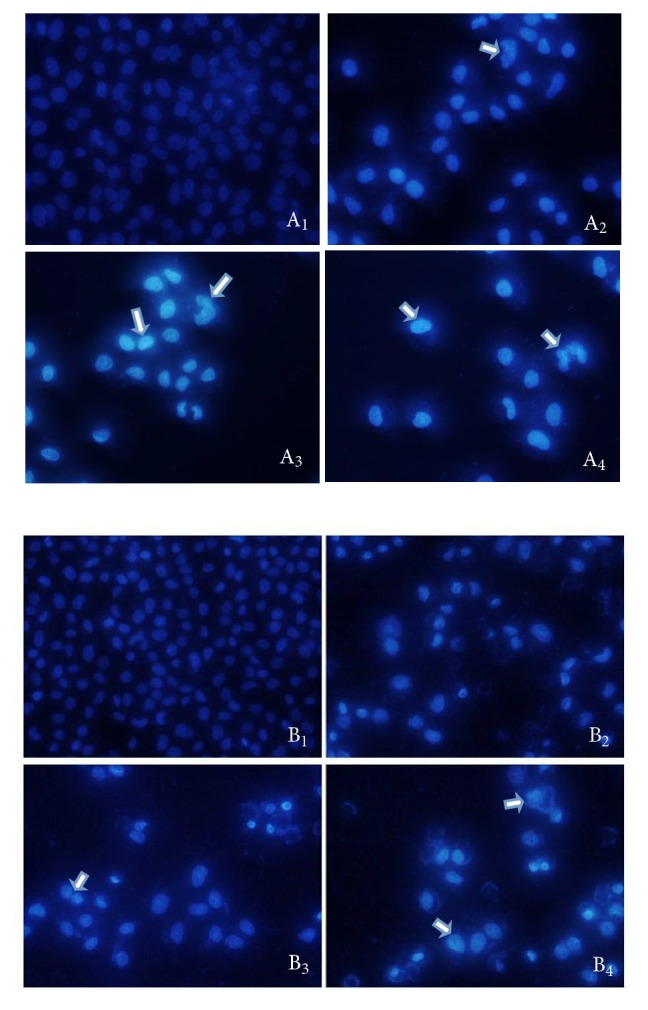
Apoptosis induced by treatment of BGC-823 (A_1_-A_4_) and SGC-7901 (B_1_-B_4_) cells with 0 (control), 20, 30, or 40 *μ*g/mL CHP for 36 h. Panels 1-4 represent treatment with 0, 20, 30, or 40 *μ*g/mL CHP, respectively.

The nuclei were stained with Hoechst 33258 to determine the incidence of apoptosis morphologically. Representative photomicrographs showed the morphological characteristics of apoptosis, such as chromatin condensation and nuclear fragmentation (indicated by arrows), in SGC-7901 and BGC-823 cells treated with different concentrations of CHP. All data were obtained within 36 h of treatment. The CHP 20, 30, and 40 *μ*g/mL groups showed significant increases in apoptosis compared with that of the control group (Figure 6), indicating that CHP increases the extent of apoptosis.

### 3.5. Flow Cytometry

**Figure 7:**
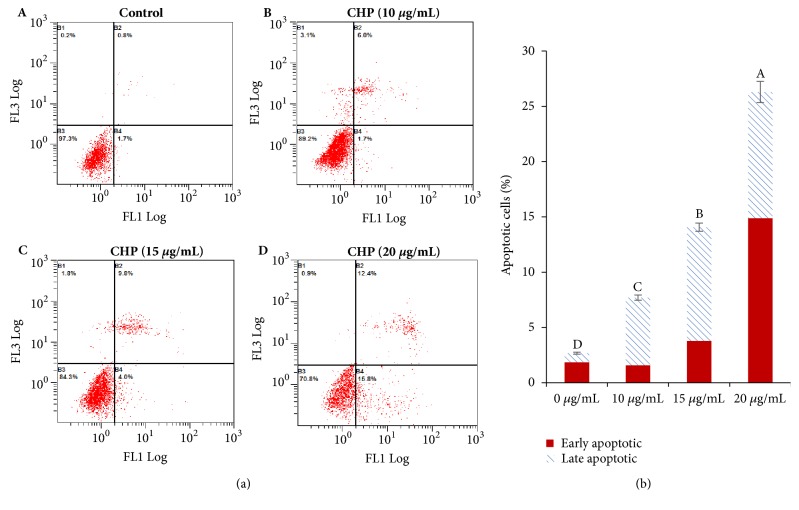
Flow cytometry analysis of SGC-7901 cell apoptosis by double staining with Annexin V-FITC and propidium iodide (PI). Values are presented as the mean ± SD of three independent experiments. Different upper case letters indicate significant difference between groups (Fisher's LSD,* P* < 0.01).

Apoptosis is frequently dysregulated during the progression of cancer [23]. After treatment of SGC-7901 cells with CHP for 24 h, flow cytometry showed that the percent of apoptotic cells was 2.67 ± 0.09, 7.7 ± 0.23, 14.07 ± 0.37, and 26.3 ± 0.95 %, for 0, 10, 15, and 20 *μ*g/mL CHP, respectively (Figure 7). Quantification of early and late apoptosis after treatment with various concentrations of CHP showed significantly increased apoptosis in the groups treated with 10, 15, and 20 *μ*g/mL CHP compared with that in the control group (*P* < 0.01).

### 3.6. CHP Inhibited In Vivo Tumour Growth in BALB/c Mice

**Figure 8:**
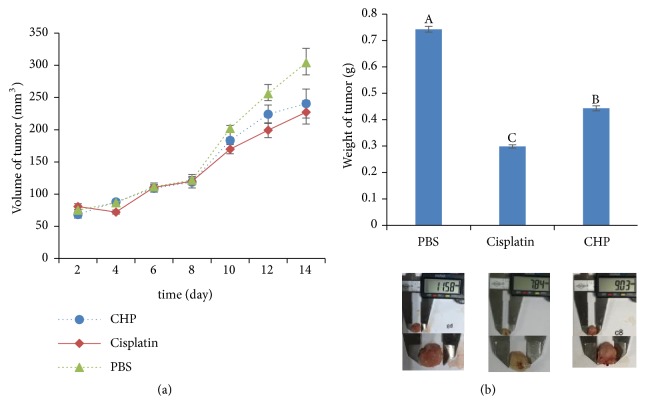
The antitumour effects of CHP* in vivo*. (a) 4T1 cells were subcutaneously injected into the BALB/c mice. Tumour volume measurements for the BALB/c mice during treatment. (b) Tumour weight at the end of treatment (Day 14). Values are presented as the mean ± SD of three independent experiments. Different upper case letters indicate significant difference between groups (Fisher's LSD,* P* < 0.01).

To elucidate whether CHP suppresses* in vivo* tumour growth, we established the live tumour model by subcutaneously transplanting 4T1 cells into BALB/c mice, which were* in situ* injection of CHP (20 mg/kg/2 d), PDD (1 mg/kg/2 d), or PBS (0.02 mL/kg/2 d). Two weeks later, the CHP-treated BALB/c mice developed smaller tumours in terms of size and weight compared with those of PBS group (Figures 8(a) and 8(b)). These results implicated that CHP can partially inhibit tumour growth derived from 4T1 cells* in vivo*.

## 4. Discussion

Malignant tumours are a major public health problem worldwide. These tumours exhibit uncontrolled proliferation, leading to death. Disorders in the control of cancer cell apoptosis play an important role in the development of cancer. The key approaches to prevent cancer are the suppression of cancer cell proliferation and acceleration of apoptosis [23]. A number of chemotherapy agents have offered significant advantages in the treatment of cancer. Owing to the advantages of high bioactivities and low toxicity, the use of natural products as a new source of anticancer agents is becoming popular [7];* A. chinensis* is such a natural source. Dried* A. chinensis* is a traditional Chinese medicine utilised to treat pain, poor digestion, and kidney diseases. Previous investigations have shown that* A. chinensis* possesses pronounced antibacterial, anticancer, analgesic, antifibrotic, and angiogenesis-promoting activities [8–10, 12]. Our previous study revealed that the anticancer active components were mainly distributed in the haemolymph and abdomen [17] and that the haemolymph from* A. chinensis* can inhibit the proliferation of human gastric cancer cell lines SGC-7901 and BGC-823, and human breast cancer cell line MCF-7 in a dose- and time-dependent manner [18–20]. Our further studies showed that the expression of caspase 3, caspase 9, and Bax proteins was significantly upregulated, and there was no significant difference in the expression of caspase 8 when SGC-7901 cells were treated with haemolymph. This indicated that apoptosis of SGC-7901 cells induced by the haemolymph might be via the mitochondrial signal pathway [24]. Although increasing evidence supports the promising anticancer effect of* A. chinensis, *several questions regarding the anticancer active components remain unanswered.

In the present study, our results demonstrated that the haemolymph extracted from* A. chinensis* inhibited the proliferation of SGC-7901 cells in a dose-dependent manner (Figure 1(a)), which was consistent with the findings of a previous study [20]. In the present study, we found that when the haemolymph was treated at 60°C, a large amount of proteins were denatured. The results showed that haemolymph proteins treated at 60°C are more effective (IC_50_ = 3.145 mg/mL) at inhibiting the proliferation of SGC-7901 cells than the untreated haemolymph (IC_50_ = 42.912 mg/mL) (Table 1), indicating the anticancer active component may be thermostable. Interestingly, the proteins identified with high confidence from the CHP of* A. chinensis* were thermostable, such as cytochrome c, ferritin, and superoxide dismutase (Table 2) [26].

In the present study, ammonium sulphate was added to the extract by gradually increasing the ratio. In the early stage of purification experiment, neutral salt can affect the nonpolar, hydrophobic, and electrostatic interactions among proteins that need to be concentrated [27]. The electrostatic charge induction on the surface of protein induces conformational change and therefore the protein cannot fully solvate to prevent aggregation. Consequently, the protein can dissolve in a low concentration of salt solution, but rarely soluble or insoluble in water [28]. The electric charge of the protein can be increased by the addition of a small amount of salts to neutralise the surface electrostatic force. The increase in salt concentration can not only neutralise the hydrostatic power on the surface of the protein, but also remove the water molecules from the protein. These water molecules are necessary for the dissolution of proteins, and the salt competes with water molecule [29]. To ensure repeatability of the results of different batches of experimental materials, a relatively wide range of 40%–60% was selected according to the results of the MTT assay (Figure 1(b)), but some target components might be inevitably lost and experimental deviation might be caused.

In the present study, two kinds of ultrafiltration membranes, 5 and 50 kDa, were used to divide the protein solution into three parts, removing most of the impurities and preparing the sample for the subsequent column chromatography. Ultrafiltration (UF) is a membrane separation process that uses a pressurised active membrane to separate particles according to their molecular weight. Under external pressure, dissolved substances and substances with pore size smaller than the membrane will be used as permeable liquid through the membrane to achieve the purpose of solution purification, separation, and concentration [30]. To improve the permeation flux of the membrane and ensure the normal and stable operation of the ultrafiltration membrane, ultrafiltration was performed after heat treatment, salting out, and preferably centrifugation at high speed to remove denatured protein from the intermediate process. The results suggest that the haemolymph proteins with intermediate molecular weight (5–50 kDa fractions) are more effective in inhibiting the proliferation of SGC-7901 cells than the low molecular weight proteins (< 5 kDa) and high weight proteins (> 50 kDa). Therefore, the 5–50 kDa fraction was selected for further purification (Figures 2(a) and 2(b)). The results of SDS-PAGE revealed that the molecular weight of the anticancer active component proteins is approximately 15 kDa (Figure 2(c)). Furthermore, most predicted molecular weights of peptides identified with high confidence were distributed from 6 to 24 kDa (Table 2), which was consistent with the results of ultrafiltration fractions (5–50 kDa).

Reversed phase chromatography is based on the hydrophobic effect between the solute, polar flow phase, and nonpolar stationary phase surface [31]. Any organic molecule has a nonpolar hydrophobic part in the structure, which is the most widely used separation mode in HPLC. The mobile phase uses acidic and low ionic strength aqueous solutions and a certain proportion of the organic modifiers such as isopropanol, acetonitrile, and methanol, which can be dissolved with water [32]. In the present study, the fraction was eluted with acetonitrile. The length of alkyl chain has no significant effect on the reverse phase retention of proteins, but the reaction of short-chain alkyl groups (such as C4 and C8) and long-chain alkyl groups (such as C18 and C22) is different in the recovery of protein activity [33]. The longer the alkyl chain is, the stronger the hydrophobicity of the stationary phase is. Therefore, it is necessary to increase the organic components of the mobile phase to rapidly elute the protein. In general, reversed phase chromatography on the alkyl-bonded silica gel is one of the methods for the separation, analysis, and purification of proteins because of its high column efficiency, good separation, and clear retention mechanism [34]. In this study, C18 column was used, and the results of MTT detection showed that the activity of antigastric cancer SGC-7901 cells was obviously enhanced after the separation and purification (Table 1).

Among the proteins identified with high confidence from the CHP of* A. chinensis *(Figure 3), Cyt c might be a noteworthy antitumour protein (Table 2), which has an intermediate role in apoptosis [23]. Previous investigations have shown that the apoptotic program can be initiated by the addition of dATP and Cyt c in cell-free extracts [35]. Once released into the cytosol, Cyt c binds to apoptotic protease activating factor-1 (Apaf-1) and procaspase-9, apoptosome was formed, and apoptosis was initiated [36]. The results of the present study demonstrated that CHP can inhibit the proliferation and increase the rate of apoptosis in gastric cancer cells (Figures 4, 5, 6, and 7). Representative microphotographs showed that morphological characteristics of apoptosis, such as chromatin condensation and nuclear fragmentation, were evident in SGC-7901 cells treated with CHP (Figure 6), and Annexin V-FITC/PI flow cytometric analysis further validated the apoptosis-inducing effect of CHP in a concentration-dependent manner (Figure 7). On account of stage IV breast cancer leads to death in most patients with breast cancer [37], and the growth and distant metastasis of 4T1-transplanted tumour model cells of breast cancer are highly similar to those of stage IV human breast cancer [38]. This animal model has been successfully applied to study drugs for the treatment of malignant tumours [39]. Moreover, the second breast in mice with the same anatomical site can better imitate the development of human breast cancer [40]. Therefore, in the present study, the 4T1 cells (1.0 × 10^6^) were inoculated subcutaneously into the second breast of each mice, and we demonstrated that CHP can suppress the growth of breast cancer 4T1 cell xenograft tumours* in vivo* (Figure 8).

Overall, to the best of our knowledge, the present study is the first to report the antiproliferative and proapoptotic effects of newly isolated and identified bioactive protein component from* A. chinensis* in cancer cells* in vitro* and* in vivo*. However, changes in apoptosis-related genes and the generation of apoptosis signals during the induction of cancer cell apoptosis require further investigation. In addition, the* in vivo* safety of these active peptides should be verified.

## Figures and Tables

**Table 1:** Summary of purification of CHP from *A. chinensis* Dallas.

No.	Purification Step	Total protein (mg)	Yield (%)	IC50 (mg/mL)
1	Haemolymph	2086.368	100	42.912

2	Heat treatment (60°C)	266.251	12.761	3.145

3	Ammonium sulphate (40-60%)	67.492	3.235	1.435

4	Ultrafiltration (5-50 kDa)	17.158	0.822	0.337

5	Semi-preparative RP-HPLC	1.162	0.056	0.026

**Table 2:** Proteins identified with high confidence from the CHP of *A. chinensis* Dallas.

Peptide sequence	Prec MW	Prec m/z	Theor MW	Theor m/z	Protein
DTLFEYLENPKK	1552.774414	777.3945	1552.777344	777.3959351	Cytochrome c
MVFAGIK	807.4334717	404.724	807.4312744	404.7229309	
LENPKK	727.4298706	364.7222	727.4228516	364.718689	
ADLIAYLEQATK	1391.730103	696.8723	1391.729614	696.8720703	
DTLFEYLENPKK	1495.751831	748.8832	1495.755859	748.8851929	
KPQERADLIAYLEQATK	2032.07959	678.3671	2032.05896	678.3602295	
VGPNLSGLIGR	1139.647217	570.8309	1139.629883	570.8222046	

EGLGIFVLDK	1090.612305	546.3134	1089.607056	545.810791	Ferritin
QNFHADSEEAINK	1501.667236	751.8409	1501.679688	751.847168	
SLGDLLTNVR	1086.604492	544.3095	1086.603271	544.30896	
VKEGLGIFVLDK	1316.761719	659.3881	1316.770386	659.3924561	
AVEAALQLEK	1070.595215	536.3049	1070.597168	536.3058472	
LAFVEIDR	977.5206909	489.7676	977.5181885	489.7663879	
TLALLHR	822.5070801	412.2608	822.5075684	412.2610474	
LFNLFYFAK	1162.605713	582.3101	1162.606323	582.3104248	

MISLCGPLSIIGR	1431.728638	716.8716	1431.757813	716.8861694	Superoxide dismutase
TIVVHADPDDLGK	1379.704834	690.8597	1378.709229	690.3618774	
VACGVIGITK	1073.583618	537.7991	1073.590332	537.8024292	
HVGDLGNICAEANGVAK	1752.820435	877.4175	1752.846436	877.430542	
YDTPELVEK	1149.551025	575.7828	1149.55542	575.7849731	
AGGQVAWTK	916.4700928	459.2423	916.4766846	459.2456055	
EAFSLFDKDGDGTITTK	1843.888916	615.6369	1843.884033	615.6352539	

LFDLFFFAK	1203.633667	602.8241	1203.632813	602.8236694	Secreted Hemocyanin-like protein

AVGVDLNLK	927.5370483	464.7758	927.5389404	464.7767334	Glutathione s-transferase

KASGQKIGQK	1044.60791	523.3112	1044.592773	523.3036499	Tyrosine-protein phosphatase corkscrew isoform x2
LVSLSEQQLIDCSR	1704.82605	853.4203	1704.835205	853.4249268	

LVSLSEQQLIDCSR	1704.82605	853.4203	1704.835205	853.4249268	Cysteine protease

INNPLK	698.3964844	350.2055	698.3963013	350.2054138	Death-associated protein 1

KLNTNSLQK	1044.60791	523.3112	1044.592773	523.3036499	Telomerase reverse transcriptase

VLHGTVVAVGPGAR	1331.76123	444.9277	1331.767334	444.929718	Heat shock protein, putative

LDEALEAAR	986.5010986	494.2578	986.5032959	494.2589111	TATA element modulatory factor

RAKEASAAEGGIK	1286.706421	644.3605	1286.694214	644.3544312	6S proteasome non-ATPase regulatory subunit 4

SILSTLYASEVMYENT	1819.820435	607.6141	1819.85498	607.6256104	ATP synthase subunit a
SLYNEFKLLTGPNNK	1736.867798	435.2242	1736.90979	435.2347107	
LLTGPNNK	856.4470825	429.2308	856.4654541	429.2399902	
INIIMNNMLNSLYNEFK	2070.905273	1036.46	2071.011719	1036.513184	
TNLFSTFDPATSIK	1541.681274	771.8479	1541.761353	771.8879395	
PFMVLIETISNLIR	1644.788452	549.2701	1644.927246	549.3164063	
STLYASEVMYE	1291.598267	646.8064	1291.564209	646.7893677	
INIIMN	716.3768921	359.1957	716.3890991	359.2018127	
MTNLFSTFDPAT	1343.644287	672.8294	1343.606812	672.8106689	

MVFIMFWFIWVR	1673.763306	837.8889	1673.86145	837.9379883	
LMYLTWK	953.5025024	477.7585	953.5044556	477.7595215	
TGLMGLLQPFADGLK	1559.709106	780.8618	1559.838135	780.9263306	
SVLGYIQLRK	1176.579468	589.297	1176.686646	589.3505859	NADH-ubiquinone oxidoreductase chain 1
IFMSFPLFF	1147.543457	574.779	1147.577637	574.7960815	
MSFPLFFCWLSSCLAETNR	2364.968506	592.2494	2365.069336	592.2745972	
SEYMNIIFM	1146.536621	574.2756	1146.508911	574.2617798	

FQGNKFYPLNK	1355.621094	678.8178	1355.687378	678.8509521	Cytochrome b
DVNNGWLMR	1104.517456	553.266	1104.502197	553.2583618	
AGRGLYYGSYK	1233.599121	617.8068	1233.614258	617.8143921	
WLWGGFSVDNATLTR	1722.773315	862.3939	1722.836548	862.4255371	
SNYDKTPFHPYF	1514.661133	758.3378	1514.682983	758.3488159	
MLGDPENFIP	1147.543457	574.779	1147.521973	574.7682495	

ISFINQNIK	1075.515259	538.7649	1075.602539	538.8085938	NADH dehydrogenase subunit 6

NDIICSQMNWK	1407.588623	704.8016	1407.627563	704.8210449	ATP synthase protein 8

TASYDREK	968.3950806	485.2048	968.4562988	485.2354431	NADH-ubiquinone oxidoreductase chain 3
TICSIISK	920.4076538	461.2111	920.5001221	461.2573242	
MMMYVMMTTI	1266.534912	634.2747	1266.519043	634.2667847	

## Data Availability

The data used to support the findings of this study are available from the corresponding author upon request.
